# A *Schmidtea nova* (Benazzi, 1982)-like Population in SW Romania (Platyhelminthes, Tricladida, Dugesiidae), a Morphological Characterization

**DOI:** 10.3390/biology15110831

**Published:** 2026-05-25

**Authors:** Anda Felicia Babalean

**Affiliations:** Department of Biology and Environmental Engineering, Faculty of Horticulture, University of Craiova, 200396 Craiova, Romania; anda.babalean@ucv.ro

**Keywords:** freshwater planarians, local population, atrial tube, functional significance

## Abstract

This study has begun as exploratory research on freshwater planarians from southwestern Romania for faunal purposes. Specimens were sampled and processed for standard histology (paraffin wax embedding, horizontal and sagittal serial sections, and Haematoxylin–Eosin staining). The histological analysis provided evidence for a population belonging to the genus *Schmidtea*. The morphological and histological characteristics were compared with those of all other *Schmidtea* species. The histological slides reveal numerous diagnostic characters for *Schmidtea nova*: the presence of two nipples on the course of the ejaculatory duct; a partly sclerotized ejaculatory duct with a wide lumen; the knee-shaped bending of the distal part of the penis papilla in most specimens; the presence of the parovarium; and the egg-shaped distension of the bursal canal musculature. Unlike typical *Schmidtea nova*, this population shows a slightly different organization of the atrial space by the presence of a muscular atrial cavity named the atrial tube here. The newly discovered population shows affinities with other *Schmidtea* species: the presence of cephalic sensory fossae, similar to *Schmidtea polychroa*, and a potential asexual reproduction mechanism by fission, similar to *Schmidtea mediterranea*. The functional significance of the atrial tube and the taxonomic status of this population are hypothesized.

## Simple Summary

*Schmidtea* is a European genus of hermaphroditic freshwater flatworms consisting of only four species: *Schmidtea polychroa*, *Schmidtea lugubris*, *Schmidtea mediterranea* and *Schmidtea nova*. Their formation during the geological eras through a complex speciation process represents the natural history of the group. The four species are morphologically distinct from each other, less in their external morphology but more so in their genital system. A new population of *Schmidtea* was discovered in the southwestern part of Romania; by the morphological characters of its copulatory apparatus, this population is closest to *Schmidtea nova* but not identical. It differs from all species of the genus by the presence in the copulatory apparatus of a muscularly delimited cavity, the atrial tube. The paper discusses the taxonomic status of this population and the possible function of the atrial tube. Regardless of its taxonomic status (a simple population of the known species *Schmidtea nova*, or a new, unnamed species), this population is part of the global metazoan diversity; its morphological presentation is valuable for more complex scientific contributions.

## 1. Introduction

*Schmidtea* Ball, 1974, is a genus of freshwater flatworms with a geographical range restricted in the West Palearctic region [1]. It was introduced into the systematics of the family Dugesiidae at the genus rank based on two morphological characters of the copulatory apparatus: the penis bulb with a double seminal vesicle, and the mixed musculature of the bursal canal [2].

After a tangled taxonomic history, it was established that the genus *Schmidtea* includes only four species: *Schmidtea polychroa* (Schmidt, 1861), *Schmidtea lugubris* (Schmidt, 1861), *Schmidtea mediterranea* (Benazzi, Baguñà, Ballester, Puccinelli, Del Papa, 1975) and *Schmidtea nova* (Benazzi, 1982). Their geographic distribution shows total or partial sympatry [1].

The biogeographic evolutionary patterns within the genus *Schmidtea* have been shown to be complex and dynamic, determined by a multitude of interconnected factors—karyological and genomic characteristics, reproductive strategies, ecological and environmental factors, and geological events [3,4,5,6,7,8,9,10,11,12].

The genus *Schmidtea* is considered monophyletic on both morphological and molecular data [13,14]. Phylogenetic relationships between the four species were for the first time resolved at the molecular level by Leria and collaborators, with the presumed synapomorphic and autapomorphic morphological characters plotted on the molecular tree [1].

Two of the four species of *Schmidtea* have been reported in Romania: *Schmidtea lugubris* [1,15,16] and *Schmidtea nova* [1]. Unidentified *Schmidtea* species are also reported: one species showing numerous similarities with *Schmidtea mediterranea* [17], the other species being closest to *Schmidtea nova* [18]. The subject of this paper is the latter species, for which additional sagittal and serial histological sections were investigated.

Molecular studies have gained exceptional prominence in recent years, with their impact and power being undeniable in species identification, species delimitation, solving phylogenetic relationships and phylogenetic reconstructions, taxonomic re-evaluations, etc., for instance, [19,20,21,22,23,24,25,26,27,28,29,30,31]. When the integrative approach is not possible, morphology remains the most accessible and important way to learn and report. Even alone, morphology is informative for function, physiology, systematics, phylogeny, at the organismal, population, species and higher taxa levels.

Because an integrative approach (molecular markers, population genetic analysis) was not possible, the main objective of this paper is the morphological characterization of the newly discovered population.

## 2. Materials and Methods

**Figure 1:**
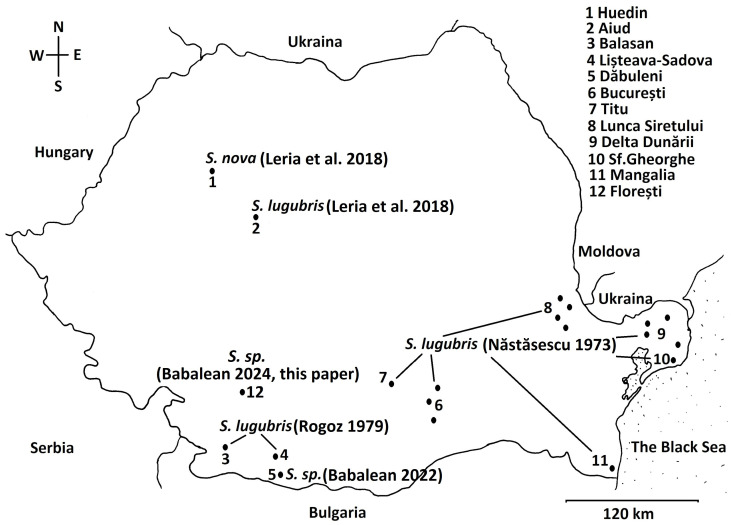
*Schmidtea* species reported in Romania [1,15,16,17,18].

The worms were collected with a paintbrush from a piped water catchment on a private property (agricultural land) in the Florești locality (44°37′08″ N 23°31′39″ E), Gorj County (SW Romania)—Figure 1. Verbal consent from the landowner was obtained for collection. The sample material is characterized as follows:

5 November 2020: Twenty-eight specimens were fixed in Beauchamp solution for 24 h and thereafter removed and stored in 70–75% ethanol. Two of the 28 specimens were prepared for usual histological serial sections: specimen labelled Fl1—42 slides in sagittal sections; specimen Fl2—12 slides in horizontal sections.

5 July 2021: Twenty-seven pieces (whole worms and fragments resulting from fission) fixed in Beauchamp solution, 2 specimens in 96% ethanol. Specimen labelled F5—69 slides in serial sagittal sections.

7 March 2022: Ten specimens fixed in Beauchamp solution, 7 specimens fixed in 96% ethanol (removed and stored in 70% ethanol), 1 specimen in absolute ethanol. Specimen labelled F1—37 slides in serial horizontal sections; specimen F2—68 slides in serial sagittal sections.

Fixation with Beauchamp solution was done using the protocol described elsewhere [32]. Beauchamp solution is a mixture of 96% ethanol:40% formaldehyde:glacial acetic acid in a volumetric ratio of 6:3:1. The fixation is performed as follows: in a crystallizer or a Petri dish, 5–6 worms are placed in a thin film of water. When the animals are well stretched, the freshly prepared fixative is quickly poured in, in a sufficient amount (I usually use 20 mL). The dish is immediately shaken vigorously, alternately clockwise and anticlockwise. In the absence of this agitation, most planarians twist. Then, each worm is carefully picked up with a pair of tweezers with a wide, thin tip (a spatula-like tweezer) and placed in another dish between 2 strips of filter paper. Two to three worms are placed between 2 strips of filter paper. For multiple fixation specimens, the strips of paper are arranged in a criss-cross pattern. A sufficient quantity of the same fixative, not the one used, but freshly prepared, is poured over the worms. After 12 h of fixation, the worms are carefully removed from the paper bandages and left free in the fixative solution. After 24 h, they are removed from the fixative and placed in 70% ethanol, where they can be stored for a long time. Fixation in absolute ethanol was done in case other researchers would like to use molecular markers.

Fixed worms were processed in a private medical laboratory by the usual method for Haematoxylin–Eosin staining: dehydration in successive ethanol baths (70% ethanol to absolute ethanol), clarification (xylol/toluol), paraffin wax embedding, sectioning at 5 μm, de-paraffining, re-hydration in successive ethanol baths (96–70%, distilled water), staining with Haematoxylin–Eosin, dehydration, clarification (xylol), and mounting of histological sections between slides and coverslips.

The histological slides will be deposited in the following museums: The Natural History Museum Grigore Antipa—Bucharest, Romania; Naturalis Biodiversity Centre—Leiden, The Netherlands; Biodiversity Museum Goettingen, Germany.

The reconstruction of the copulatory apparatus was achieved by tracing (drawing) the photographs of the serial histological sections on transparent paper. All the microphotographs were taken by the author with a Levenhuk microscope digital camera, M1400 PLUS, on a Karl Zeiss Jena binocular; calibration with Kalibrácios tárgylemez MikRet51 (Budapest Távcsö Centrum). The image quality in the figures was not improved, just adjusted for contrast and brightness. The images were minimally processed with the Paint (Microsoft Windows) 22H2 and GIMP 3.0.4 programmes for writing the letters and for scaling to 300–600 dpi.

GenAI was not used in this work.

Abbreviations used in the figures: at—atrial tube; b1—the first penis bulb with the primary seminal vesicle; b2—the second penis bulb with the secondary seminal vesicle; bc—bursal canal; ca—the common atrium; cb—copulatory bursa; egg—egg-shaped expansion of the bursal canal musculature; ej—ejaculatory duct; go—gonopore; ma—male atrium; np—nipple; od—oviduct; ov—ovary; pb—penis bulb; ph—pharynx; pov—parovarium; pp—penis papilla; psv—primary seminal vesicle; sg—shell glands; spv—spermiducal vesicles; ssv—secondary seminal vesicle; tb—ovarian tuba; te—follicular testes; vd—vas deferens.

## 3. Results

### 3.1. Systematics

Order Tricladida Lang, 1881

Suborder Continenticola Carranza, Littlewood, Clough, Ruiz-Trillo, Baguñà, Riutort, 1998

Family Dugesiidae Ball, 1974

Genus *Schmidtea* Ball, 1974

*Schmidtea* sp.

### 3.2. Morphology

**Figure 2:**
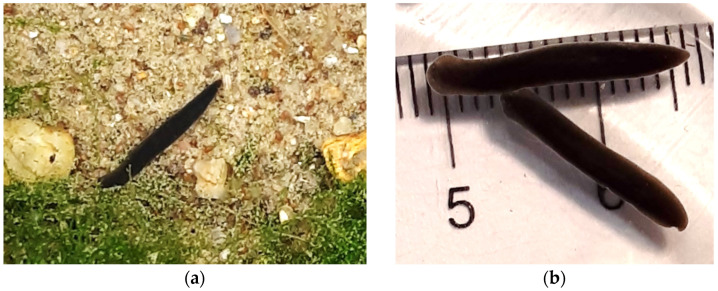
Living specimens of *Schmidtea* sp.: (**a**) in nature; (**b**) in laboratory.

**Figure 3:**
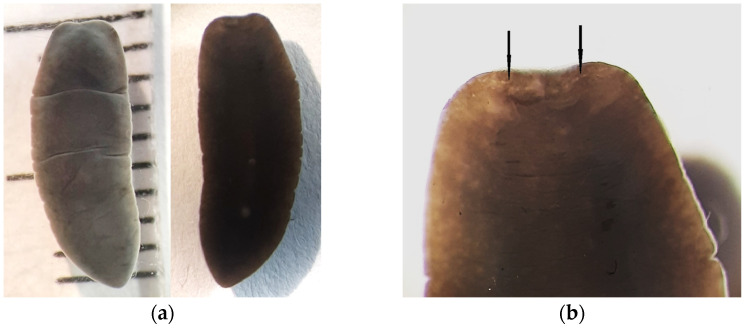
*Schmidtea* sp. fixed specimens: (**a**) dorsal and ventral view; (**b**) head with sensory fossae indicated by arrows.

**Figure 4:**
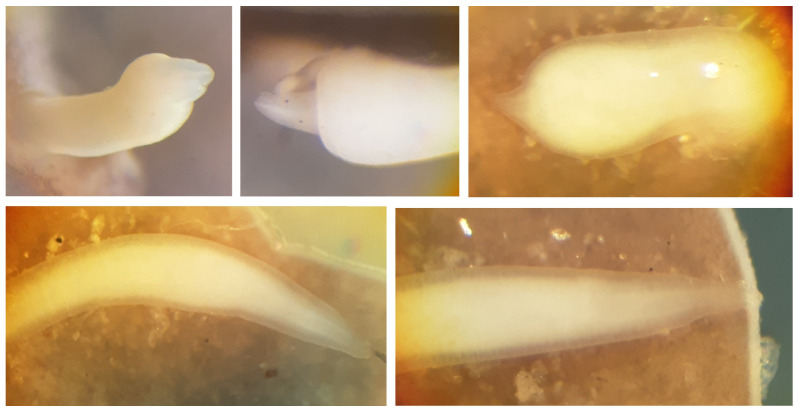
The aspect of the protruded penis papillae in five specimens.

Living specimens measure up to 18–19 mm long. The colour is black dorsally and dark brown to black ventrally—Figure 2 and Figure 3. The head is obtusely pointed or rounded, the auricular region is barely marked, and the neck region is indistinctive. Two eyes are set in pigment-free patches, placed very close to the anterior margin of the head. Numerous specimens show only the pigment-free patches, but no pigmented eye. One specimen had 4 eyes (supernumerary eyes). The ventral margin of the head presents whitish and luminous spots, which are interpreted as sensory fossae—Figure 3b. Numerous specimens fixed in ethanol show protruded penis papillae of variable length and shape (straight or bent)—Figure 4.

Pharynx short and located in the second half of the body; the musculature of the planariid type, cf. Sluys [33].

**Figure 5:**
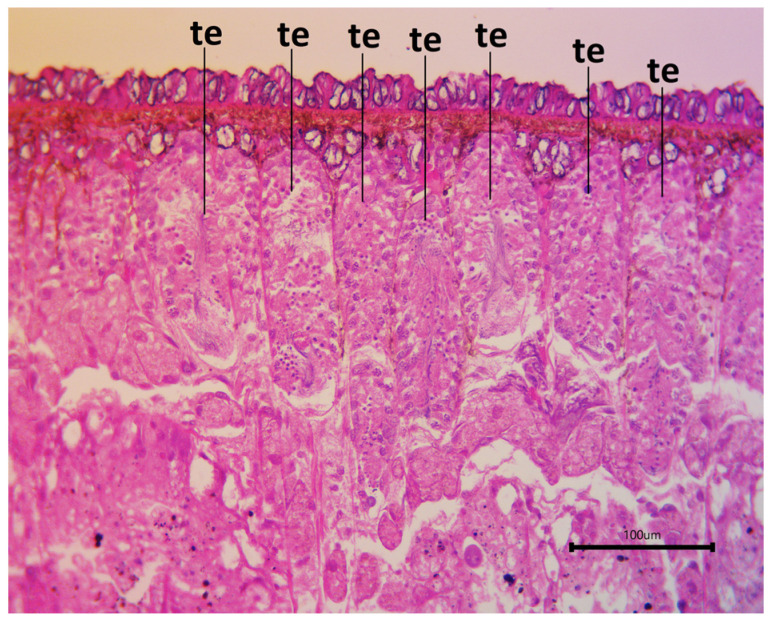
Aspect of the follicular testes indicated by arrows, in specimen F2-16-2 (specimen F2, slide 16, section 2); scale bar 100 µm.

**Figure 6:**
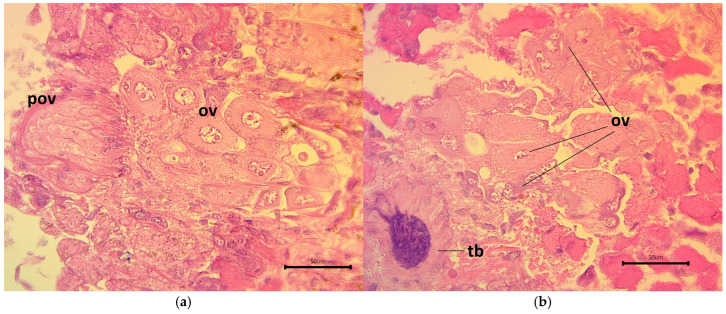
The ovary: (**a**) ovary and par-ovarium in specimen F2-27-2; (**b**) ovarian tuba and different types of ovarian cells in specimen F1-10-2; scale bar 50 µm.

Follicular testes present in all specimens, situated dorsally, on lateral sides of the body, throughout the body length. The intercommunication between the follicles is rather indistinctive—Figure 5. Ventral ovaries present in all specimens, showing the parovarium—Figure 6a; the ovarian tubas show a highly basophilic content, which may represent spermatozoa—Figure 6b.

**Figure 7:**
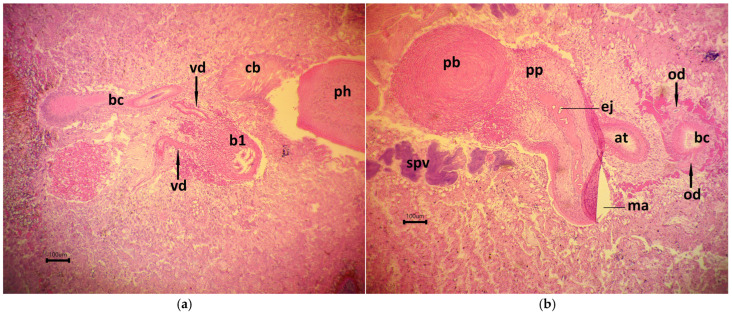
The entrance of the gonoducts: (**a**) the entrance of the vasa deferens into the primary penis bulb, section F1-30-1, anterior to the right; (**b**) the entrance of the oviducts into the bursal canal, section F1-15-2, anterior to the left; scale bar 100 µm.

**Figure 8:**
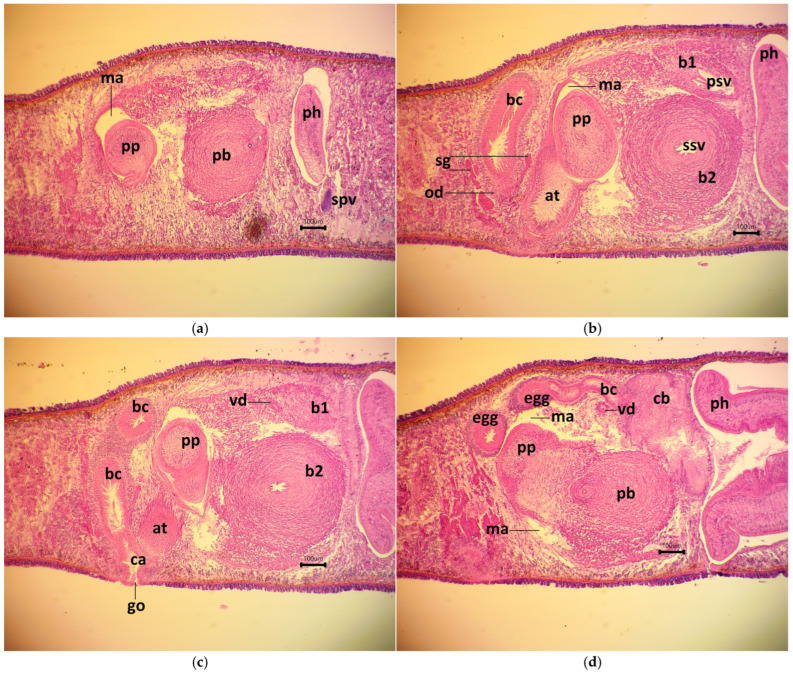
Microphotographs revealing the anatomy of the copulatory apparatus in specimen F5, sagittal sections: (**a**) section F5-26-2; (**b**) section F5-30-1; (**c**) section F5-31-1; (**d**) F5-34-2; anterior to the right; scale bar 100 µm.

**Figure 9:**
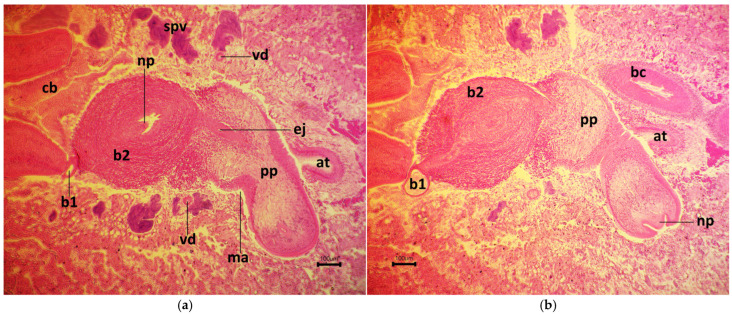
The nipples along the ejaculatory duct: (**a**) specimen F1-17-3; (**b**) specimen F1-20-2; anterior to the left; scale bar 100 µm.

**Figure 10:**
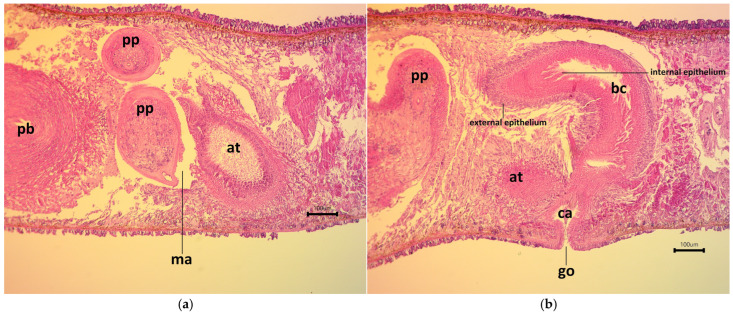
Characteristics of the atrial tube, penis papilla, bursal canal and common atrium: (**a**) section F2-33-4; (**b**) section F2-29-1; anterior to the left; scale bar 100 µm.

**Figure 11:**
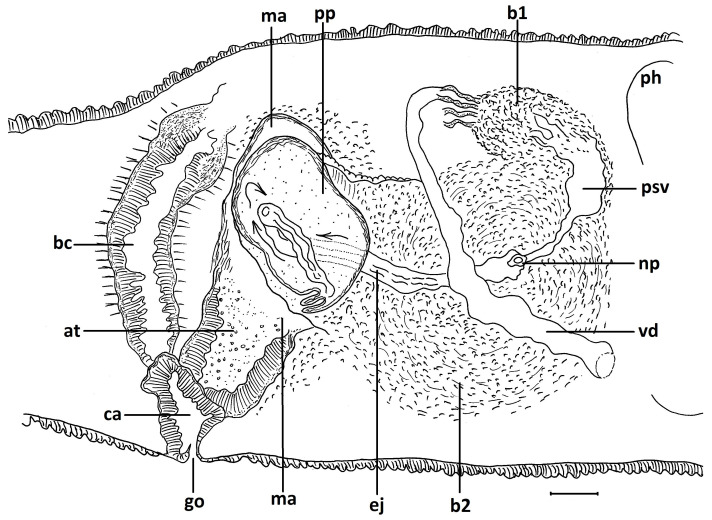
Sagittal reconstruction of the copulatory apparatus—the male component highlighted, specimen F5; anterior to the right; scale bar 100 µm.

**Figure 12:**
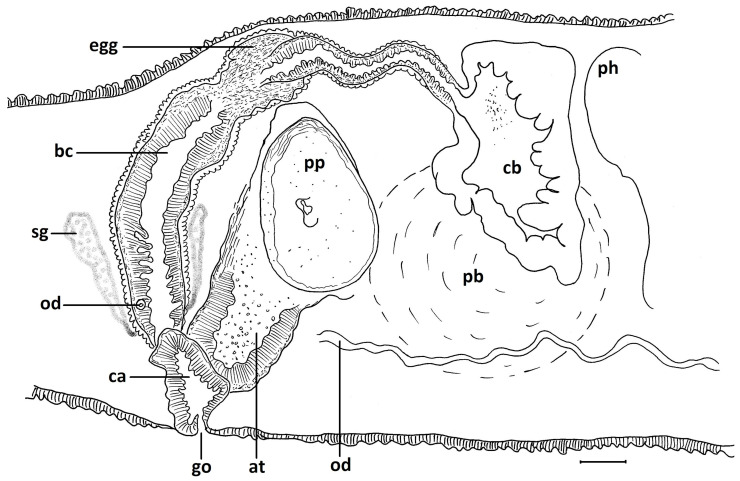
Sagittal reconstruction of the copulatory apparatus—the female component highlighted, specimen F5; anterior to the right; scale bar 100 µm.

The copulatory apparatus—Figure 7, Figure 8, Figure 9, Figure 10, Figure 11 and Figure 12.

The vasa deferentia open separately into the primary seminal vesicle—Figure 7a; at the level of the pharynx, they form very large spermiducal vesicles with a highly basophilic aspect—Figure 7b, Figure 8a and Figure 9.

The penis bulb is a very well-developed muscular mass, consisting of two regions (bulbs) with a double seminal vesicle: (i) the first bulb with the anterior primary seminal vesicle (psv) surrounded by a wall of an internal thin layer of circular musculature and an external layer of irregular musculature and (ii) the second bulb with the posterior secondary seminal vesicle (ssv) surrounded by a thicker wall consisting of concentric layers of musculature—Figure 8, Figure 9 and Figure 11. The two regions of the penis bulb are interconnected by a muscular constriction with a narrow duct (lumen) that is part of the ejaculatory duct. It opens through a diaphragm (nipple) into the secondary seminal vesicle; a second diaphragm is found at the tip of the ejaculatory duct and penis papilla—Figure 9.

The penis papilla, when long, is bent apically in most specimens.

**Figure 13:**
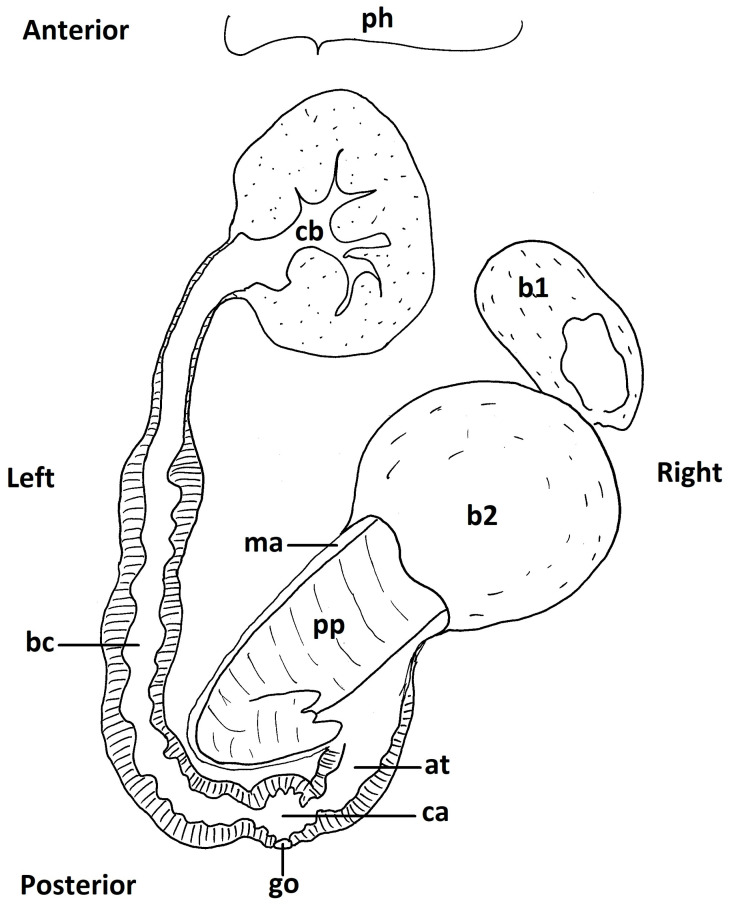
Schematic representation of the position of the components of the copulatory apparatus, dorsal view, inferred from the histological slides.

**Figure A1:**
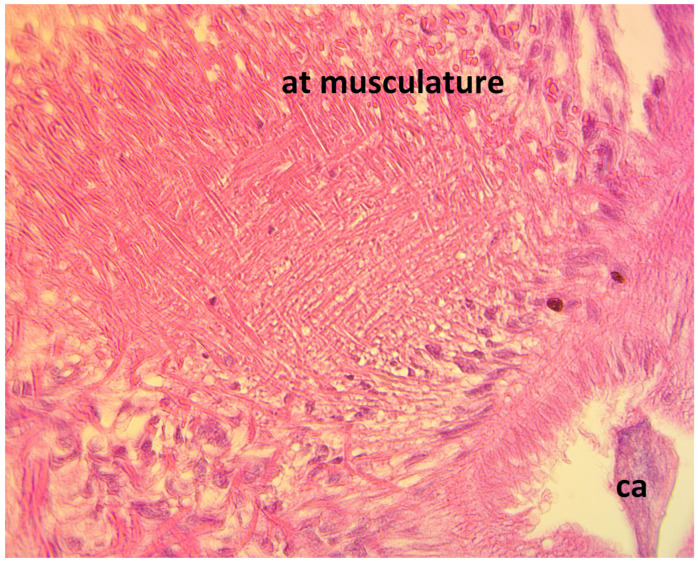
The disposition of the muscular fibres in the wall of the atrial tube, and epithelium and musculature of the common atrium, section F2-29-1.

From the outside inward, the histology of the penis papilla is characterized by a tall epithelium at the base of the papilla, which becomes low towards the tip, where it is no longer visible; the muscular layer is predominantly composed of circular fibres; under the muscular layer there is a filling tissue that looks like loose fibrous tissue with rare muscle fibres, richer in cells towards the tip of the papilla; centrally, there is the ejaculatory duct lined with very flat cells (endothelial type) and surrounded by a layer of more basophilic cells, probably secretory cells that produce a secretion to be sclerotized—Figure 7b, Figure 8, Figure 9 and Figure 10. The penis papilla occupies the male genital atrium. In some specimens, the tip of penis papilla and the opening of the ejaculatory duct remain enclosed in the male genital atrium. The ejaculatory duct does not open directly into (towards) the hermaphrodite atrium, as would be expected, for the evacuation of sperm. The male genital atrium communicates with a ventral large muscular cavity, here named the atrial tube, similar in aspect with the bursal canal in terms of diameter and having a muscular wall—Figure 7b, Figure 8b,c, Figure 9, Figure 10, Figure 11 and Figure 12. The wall of the atrial tube appears to be formed by intermingled musculature—Figure 8c, Figure 10b and Figure A1. The atrial tube has two openings: (i) a wider opening into the male atrium, at a different level than the tip of the penis papilla (the tip of the ejaculatory duct), and (ii) a narrow opening, near the opening of the bursal duct, into a small ventral cavity bearing the gonopore—Figure 8c, Figure 10b, Figure 11 and Figure 12 (to highlight the wall of the common atrium, the openings of the atrial tube and bursal canal into the common atrium are not drawn in Figure 11 and Figure 12). This small ventral cavity is considered by the author to be the true common hermaphroditic atrium. The cavity of the common atrium is lined with a tall, secretory epithelium separated from the mesenchyme only by a very thin layer of long muscle fibres—Figure 8c, Figure 10b and Figure A1. In the common atrium, the atrial tube opens on the right side, and the bursal canal opens on the left—Figure 13.

The copulatory bursa (with spermatophore in specimen F5) has a dorsal position, under the pharynx—Figure 7a, Figure 8d, Figure 9a and Figure 11. The bursal canal runs posteriorly over the penis, takes a descending, non-angled course coming posterior to the male atrium and opens into the common genital atrium—Figure 8c, Figure 10b and Figure 12. The bursal canal has a very wide lumen surrounded by a wall of intermingled musculature. The wall of the bursal canal has an uneven thickness along its length, which is thinner in the half part towards the bursa and thicker in the half towards the common atrium. In the latter region, the wall musculature forms 1–2 egg-shaped expansions—Figure 8, Figure 9b, Figure 10b and Figure 12. The bursal canal is lined with a tall secretory epithelium; externally, it is covered by a pseudostratified epithelium (multi-stratified appearance)—Figure 10b.

The two oviducts open separately into the bursal canal, close to its entrance into the hermaphrodite atrium—Figure 7b and Figure 12.

Shell glands—abundant in the distal part of the bursal canal—Figure 8b and Figure 12.

### 3.3. Aspects of Reproductive Biology

Asexual maturity was assessed by the presence of the gonopore as an indication of sexual maturation capacity. The distribution of sexual and asexual individuals in the three sampling periods is as follows.

November: asexual: 12 small and very small specimens (1–3 mm), few with traces of fission and regeneration after fission. One 3 mm long specimen shows a tendency to sexualize, with a barely visible gonopore in formation. Most of the asexual specimens (7 specimens) were intact, suggesting that they arose by hatching. Sexual: 16 intact specimens with clearly visible gonopores. November specimens are the smallest.

March: all the specimens are sexual; they are the largest size.

**Figure 14:**
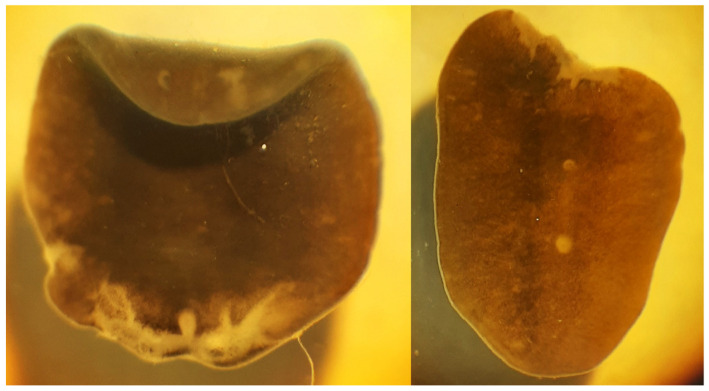
Fragments of worms resulting from fission.

July: only one asexual, intact specimen, with no trace of fission; 16 intact sexual specimens of medium and large size; 11 asexual and sexual fragments. The pieces resulting from fission are of various types: only the head with eyes and no orifices (mouth and gonopore), the body with both orifices (mouth and gonopore), and body with head and only one orifice (mouth)—Figure 14.

Fission appears to occur more intensely in July, but it is unclear whether the fission is physiological (natural, true sexual reproduction) or is produced by injuries during sampling.

## 4. Discussion

### 4.1. Morphology

The histological slides and resulting photographs were compared with the photographs provided by Leria et al. [1].

By the mixed musculature of the bursal canal, the newly described population is assigned to the genus *Schmidtea*. Following the diagnostic characters given by Leria et al. [1], this population most closely resembles *Schmidtea nova* by the following morphological characters: the presence of two nipples (diaphragms) on the course of the ejaculatory duct; a partly sclerotized ejaculatory duct with a wide lumen; the knee-shaped bending of the distal part of the penis papilla in most specimens; the presence of the parovarium; and the egg-shaped distensions of the bursal canal musculature.

The atrial tube can be interpreted (described in words) differently—either as a hermaphroditic atrium (because it communicates with the male atrium), or as an additional cavity that opens next to the bursal canal into a small ventral space (cavity) bearing the gonopore, the true hermaphroditic atrium. Regardless of the interpretation, the atrial tube is a distinct, muscularly delimited cavity that communicates with the male atrium at a level other than the tip of the penis papilla (and the opening of the ejaculatory duct).

The *Schmidtea* sp. population presented in this paper shows similarities with other *Schmidtea* species, including *Schmidtea polychroa* based on the presence of sensory fossae, as presented by DeVries & Sluys [2]. Possible reproduction by fission would bring it closer to *Schmidtea mediterranea* [34,35].

### 4.2. Functional Interpretation of the Atrial Tube

The functional significance of the atrial tube can at most be suggested, in the context provided by the literature. Several aspects of fertilization biology in freshwater planarians are not covered enough or at all by the literature, including proterandry and protogyny; the way and route by which spermatozoa reach the ovarian tuba or the ovary from the copulatory bursa/bursal canal; the stage of the oocyte at the time of fertilization (when the spermatozoon fuses with the oocyte): the primary oocyte (diploid—2n); metaphase I, metaphase II, or meiosis completed.

The freshwater planarians are hermaphroditic with cross fertilization and sperm exchange by the reciprocal insertion of the penis papilla into the bursal canal of the partner [36,37]. Oocyte fertilization was reported to take place in the ovarian tuba [36] or inside the ovaries in *Schmidtea polychroa* [38], while Sluys has attributed to the ovarian tuba the function of resorption of sperm excess [39]. After 1–2 weeks after copulation, the eggs are released from the ovary, and they are fertilized as they pass through the ovary tuba on their way to the genital atrium [36]. Several fertilized eggs together with yolk cells are enclosed in a cocoon secreted by cells surrounding the genital atrium [36].

In the author’s opinion, the presence of spermatozoa inside the ovary or the ovarian tuba is not strong evidence for cross-fertilization. The spermatozoa inside the ovary or tuba may come from the testes of the same individual, in which case a mechanism of avoiding self-fertilization should exist. The cause of spermatozoa presence in the ovarian tuba could be related not only to resorption of sperm excess but also to the development of oocytes (development induction), in one of the stages of the oocyte lineage.

In any case, the role of the atrial tube as an atrial space must be related to sexual reproduction. It can be hypothesized that the atrial tube is the site of fertilization or the storage site of the cocoon until expulsion.

### 4.3. The Evolutionary Significance of the Atrial Tube

The evolutionary significance of the atrial tube for the genus *Schmidtea* and for assigning a taxonomic status to this newly discovered population is unknown—it may be either a lost character during evolution or an acquired character (an acquisition, a recent character); it may also have no phylogenetic importance. I see the following scenarios (hypotheses).

(A) If this is a lost character, the newly described population belongs to an old species, that is, a good species—a new species for science, to be named. This species gave rise to *Schmidtea nova* by the loss of the atrial tube. Its very small range (so far, a single collection point) should not be a counterargument to this hypothesis. On the one hand, additional sampling over a very large area (at least Europe and even adjacent areas) might show its presence in other areas as well. On the other hand, if this population represents an old and good species, its distribution area could have been drastically reduced through various mechanisms—competition with other species, climatic factors, etc.

(B) If this is an acquired character, it means that *Schmidtea nova* gave rise to this morphologically distinct population that might be a subspecies or a good species—a new species for science, to be named.

**Figure A2:**
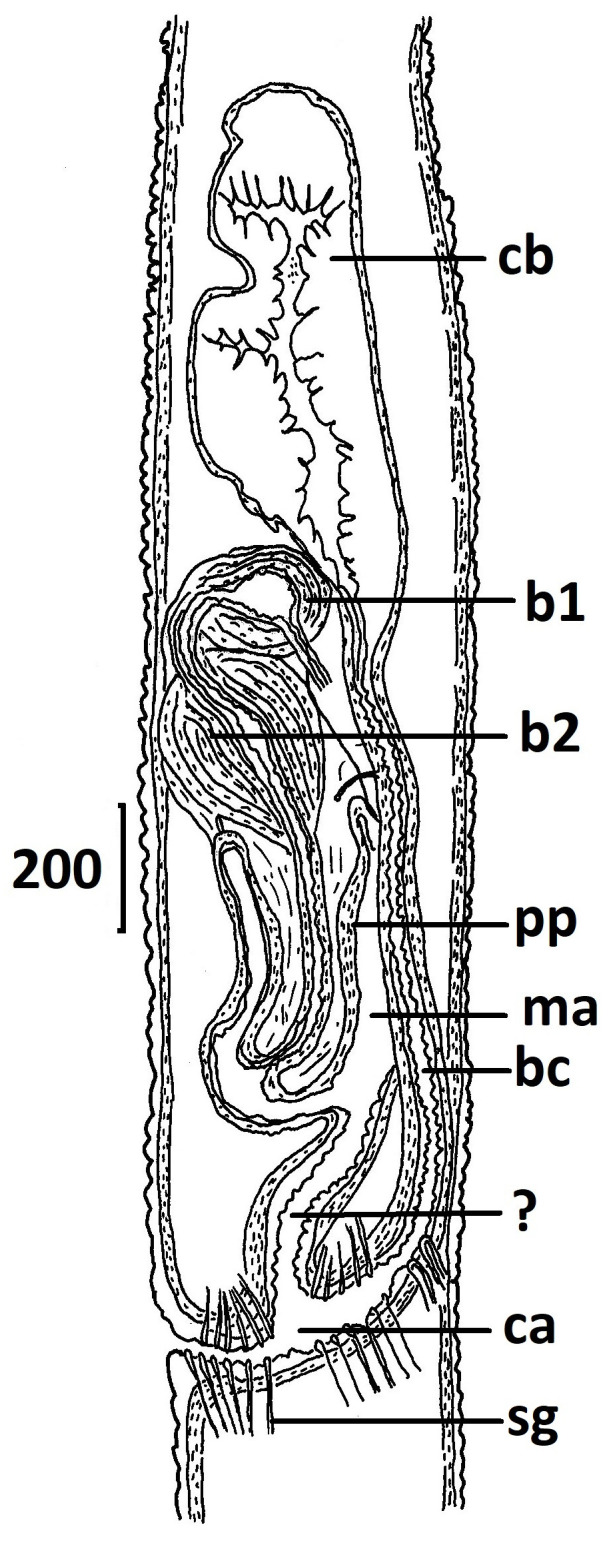
Sagittal reconstruction of the copulatory apparatus of *Schmidtea lugubris* from Sf. Gheorghe Pool, scale bar 200 µm, redrawn from Năstăsescu [15], “?”: unknown structure.

It is worth mentioning the variation in the atrial space morphology in different populations of *Schmidtea lugubris* from the Danube Delta [15]. Specifically, the population from Balta Sfântu Gheorghe (Sfântu Gheorghe Pool) has the male atrium and the hermaphrodite atrium clearly separated. The author’s drawing [15] (p. 206, Figure 4) shows a tubular connection between the two atria, a narrow and quite long tube—Figure A2. What does this tube represent? Is it an atrial tube or an atrial tube precursor? What is the real identity of the population from Sfântu Gheorghe Pool? Would this population still be assigned to the species *Schmidtea lugubris* using modern molecular tools?

## 5. Conclusions

The taxonomic status of the newly described *Schmidtea* species is unknown. By the presence of the atrial tube, this population appears to be different from all other *Schmidtea* species and distinct. Therefore, this morphological entity is a candidate for being a new species for science. Due to its many similarities to *Schmidtea nova*, this population could be a subspecies and therefore a candidate to become a new species during the evolutionary process. Nevertheless, to achieve the subspecies rank, it would have to demonstrate the condition of a larger range. For now, it can only be considered a local population of an unidentified *Schmidtea* species. An integrative taxonomic approach is necessary to establish the taxonomic status of the newly presented population and also to confirm the taxonomic status of the other four *Schmidtea* species.

The need for a comprehensive reinvestigation of the genus *Schmidtea* is obvious; it should cumulatively and simultaneously answer several questions—species composition, phylogenetic relationships, the timing of species emergence. Such a reinvestigation can only be done in an integrative manner, using samples from a very large area.

Limitations of the study include too few specimens and a lack of integrative taxonomic approaches.

## Data Availability

The original contributions presented in this study are included in the article and the link: https://www.biorxiv.org/. Further inquiries can be directed to the author.
